# Cone beam computed tomographic evaluation of infraorbital canal protrusion into the maxillary sinus and its importance for endoscopic surgery

**DOI:** 10.1016/j.bjorl.2022.07.002

**Published:** 2022-08-01

**Authors:** Gozde Serindere, Mehmet Serindere

**Affiliations:** a Hatay Mustafa Kemal University, Faculty of Dentistry, Department of Dentomaxillofacial Radiology, Hatay, Turkey; b Hatay Education and Research Hospital, Department of Radiology, Hatay, Turkey

**Keywords:** Anatomy, Cone beam computed tomography, Infraorbital canal, Maxillary sinus

## Abstract

**Objective:**

The aim of this retrospective study is to investigate the prevalence of Infraorbital Canal Protrusion (ICP) degree into the maxillary sinus and its relationship with variations in adjacent structures on Cone Beam Computed Tomography (CBCT) images.

**Methods:**

350 CBCT images (700 Infraorbital Canal [IC]) were evaluated retrospectively. ICP was divided into 3 subtypes according to the protrusion degree. The correlation between IC types and variations in adjacent anatomical structures (Haller cell, middle nasal concha pneumatization, maxillary sinus mucosal thickening and septa) was evaluated. The distance between Infraorbital Canal and Cnine Root (IC-CR) was also measured. For type 3, measurements were performed on IC as the length of the bony septum from the IC to the Mxillary Sinus Wall (IC-MSW), the distance from the inferior orbital rim, where the IC begins to protrude into the maxillary sinus (IOR-ICP), the vertical distance from the IC to the Maxillary Sinus Roof (IC-MSR) and Floor (IC-MSF).

**Results:**

The prevalence of type 1, 2 and 3 was 62.9%, 29.1%, and 8% respectively. IC-CR was 10.2, 10.7 and 11.4 mm in type 1, 2 and 3, respectively. IC-MSW, IOR-ICP, IC-MSR and floor IC-MSF was 3.8, 10.9, 7.4 and 27.7 mm, respectively. On the right and left side, statistically significant correlation was found between IC types and the presence of the Haller cell and sinus septa. But there was no significant correlation between IC types and middle concha pneumatization.

**Conclusion:**

Accurate diagnosis of ICP is very important in preventing infraorbital nerve damage in surgical procedures to be performed in the maxillary region. The results of this study could be a guide for surgical planning in this region.

**Level of evidence:**

Retrospective study.

## Highlights

• Accurate diagnosis of ICP is very important in preventing infraorbital nerve damage in endoscopic sinus surgery.
• Ignoring the ICP may cause undesirable results during endoscopic sinus surgery.
• Therefore, in all conditions, maximum attention should be paid to anatomical variations in surgical procedures.

## Introduction

The infraorbital nerve is the terminal branch of maxillary nerve which in turn is the second branch (V2) of the trigeminal nerve. It leaves the inferior orbital fissure to enter the orbit through the pterygopalatine fossa. The nerve travels through the Infraorbital Canal (IC) along the orbital floor and normally exits through the infraorbital foramen of the maxillary bone with the inferior orbital vein and artery.^1^, ^2^ Infraorbital nerve is responsible for the sensory innervation of the following structures: upper cheek skin, maxillary sinus mucosa, teeth from maxillary incisors to premolars, sometimes mesiobuccal root of first molar tooth and neighboring buccolabial gingiva and periostium, the skin and conjunctiva of the inferior eyelid, part of the nose, and the skin and mucosa of the upper lip.^3^

Variation in course may cause the Infraorbital Canal Protrusion (ICP) from the infraorbital foramen to maxillary sinus.^4^, ^5^

The increasing degree of the ICP may result in iatrogenic infraorbital nerve damage during surgical procedures that manipulate or reconstruct the orbital floor.^6^ For this reason, having an exact knowledge of the anatomic variations and the morphological characteristics of the IC is critical for surgeons. Preoperative radiological assessment of the IC corpus types is necessary for surgical operations of the orbital floor reconstruction, regional ION block and radiofrequency ablation neurotomy in V2 trigeminal neuralgia.^7^ Additionally, functional endoscopic sinus surgery is one of the most common operations in Otorhinolaryngology, which can cause many complications.^8^

Today, Cone Beam Computed Tomography (CBCT) imaging is an accepted method for radiological assessment of paranasal sinuses and infraorbital foramen and IC due to its low radiation dose, high bone resolution and easy image processing.^9^, ^10^

Because of the importance of this anatomical structure, this retrospective study was planned to investigate the prevalence of ICP into the maxillary sinus and its relationship with variations in adjacent structures on CBCT images.

## Methods

This study was approved by Ethical Committee of the Hatay Mustafa Kemal University (decision date and number: 01/07/2021 and 15) and was in accordance with the principles of the Helsinki Declaration.

CBCT images of patients who applied to the Department of Dentomaxillofacial Radiology, were selected from the database. No radiographs were taken for this study. The CBCT images taken for any reason (impacted teeth, jaw cysts and tumors, etc.) were included in the study and analyzed retrospectively. All CBCT images were obtained using CBCT OP 3D Pro machine (KaVo, Germany) with a field of view varying from 5 × 5 cm to 13 × 15 cm diameter (operating parameters: 90 kV, 5 mA, 8.14 s of exposure time, 0.38 mm voxel size).

Based on the classification made by Ference et al.,^11^ the IC was divided into 3 types according to the degree of protrusion:

Type 1 ‒ The IC is located entirely within the roof of the maxillary sinus.

Type 2 ‒ IC is located under the roof of the sinus, but remains adjacent to it.

**Figure 1:**
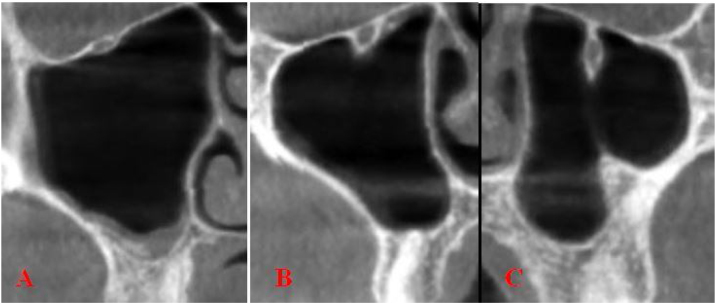
Coronal scans of Type 1 (A) and Type 2 (B) and Type 3 (C) based on the classification made by Ference et al.^11^

Type 3 ‒ The IC descends into the sinus lumen, suspended from the sinus roof within a septa or lamella of the infraorbital ethmoid cell (Fig. 1).

The morphological measurements based on the study of Haghnegahdar et al.^12^ and Kalabalık et al.^13^ were as follows:

Distance between the Infraorbital Canal and Canine Root (IC-CR): This measurement was performed for all types of ICP. A line perpendicular to the canine crown was drawn in the coronal plane, and the maximum horizontal distance from the center of the IC to this line was measured in the axial plane.^12^

**Figure 2:**
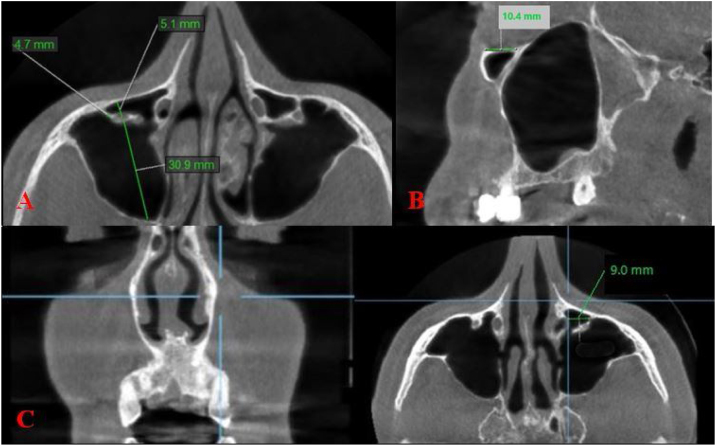
Measurements performed in the study.

For Type 3 IC:

- The maximum length of the bony septum from the IC to the maxillary sinus wall was measured on the axial images (IC-MSW);
- The distance from the inferior orbital rim, where the IC begins to protrude into the maxillary sinus, was measured on parasagittal images (IOR-ICP);
- Maximum vertical distance from the IC to the Maxillary Sinus Roof (IC-MSR) and from the IC to the maxillary sinus floor (IC-MSF) were measured on the coronal images^13^ (Fig. 2).

**Figure 3:**
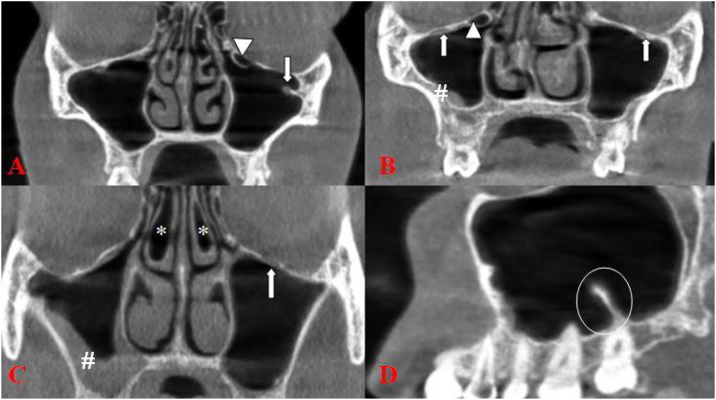
(A) Type 3 (arrow) with Haller cell (arrowhead) (B) Type 1 (arrow) with Haller cell (arrowhead) and mucosal thickening (hashtag) (C) Type 1 (arrow) with bilateral middle nasal concha pneumatization (asteriks) and mucosal thickening (hashtag) (D) Maxillary sinus septa (circle).

Haller cell, presence of middle nasal concha pneumatization, septa and mucosal thickening in the maxillary sinus were also examined in the images.^5^, ^13^, ^14^ Mucosal thickening was recorded when the mucosal thickness exceeded ≥3 mm in the sinus.^13^, ^14^ (Fig. 3). Measurements were performed by two observers who had 6 years (M.S and G.S) clinical experience in general radiology and dentomaxillofacial radiology. All evaluations and measurements were performed on a 15.6-inch full HD notebook monitor with resolution of 1920 × 1080 pixels.

SPSS version 22 software (SPSS Inc., Chicago, IL, USA) was used to enter and analyze data. Descriptive analysis was performed by evaluating categorical variables belong to numbers and percentages (%), and their mean and standard deviation values. Inter-observer agreement was performed by calculating Cronbach’s alpha for internal consistency. Cronbach’s alpha values greater than 0.7 was the minimum acceptable level for internal consistency.^15^ The data were evaluated statistically by the Chi-Square test; *p* < 0.05 were considered statistically significant.

## Results

A total of 700 Infraorbital Canals (ICs) (350 right, 350 left) were analyzed. One hundred and seventy-seven (50.6%) of the 350 patients were males and 173 (49.4%) were females. The ages of the patients ranged between 18 and 87, with a mean age of 36.5 years. There was high internal consistency (Cronbach’s alpha > 0.7), indicating high reliability for each scale.

The prevalence of type 1, 2 and 3 IC was 62.9%, 29.1% and 8% respectively. The number of type 1 was higher both in females and males. The mean IC-CR was 10.2, 10.7 and 11.4 mm in type 1, 2 and 3, respectively. The prevalence of the presence of Haller cell, mucosal thickening, pneumatization of middle concha and maxillary sinus septa was 21.7%, 57.1%, 37.7% and 36.9%, respectively. The detailed descriptive statistics information was showed in Table 1.

**Table 1:** Descriptive statistics information.

Variables	n	Minimum	Maximum	Mean	SD
Age	350	18	87	36.5	16.9
CR-IC (Right)	350	4.1	19.2	10.6	2.5
CR-IC (Left)	350	4.3	18.7	10.2	2.4

	Frequency	Per cent
Gender		
Female	173	49.4
Male	177	50.6
Protrusion Right		
1	225	64.3
2	99	28.3
3	26	7.4
Protrusion Left		
1	215	61.4
2	105	30.0
3	30	8.6
Haller cell		
0	274	78.3
1	76	21.7
Mucosal thickening		
0	150	42.9
1	200	57.1
Middle nasal concha pneumatization		
0	218	62.3
1	132	37.7
Sinus septa		
0	221	63.1
1	129	36.9

Except type 1 and 3 of right side, no significant difference was found between the other types of right and left sides and gender (*p* > 0.05).

The prevalence of the presence of Haller cell, mucosal thickening, pneumatization of middle concha and maxillary sinus septa was 21.7%, 57.1%, 37.7% and 36.9%. On the right side, statistically significant correlation was found between IC types and the presence of the Haller cell and sinus septa (*p* < 0.05). But there were no statistically significant correlations between IC types and the presence of mucosal thickening and middle concha pneumatization (*p* > 0.05). On the left side, statistically significant correlation was found between IC types and the presence of the Haller cell, mucosal thickening and sinus septa (*p* < 0.05). But there were no statistically significant correlations between IC types and the presence of middle concha pneumatization (*p* > 0.05) (Table 2).

**Table 2:** Correlation of ICP types with the neighbouring structures.

	Right-protrusion types			*p*-value
	1	2	3	
	Frequency (%)	Frequency (%)	Frequency (%)	
Haller cell	40(52.6)	24(31.6)	12(15.8)	0.000
Mucosal thickening	130(65)	56(28)	14(7)	0.8
Middle nasal concha pneumatization	86(65.2)	35(26.5)	11(8.3)	0.6
Sinus septa	70(54.3)	40(31)	19(14.7)	0.000

	Left-protrusion types			*p*-value
	1	2	3	
	Frequency (%)	Frequency (%)	Frequency (%)	
Haller cell	38(50.0)	25(32.9)	13(17.1)	0.000
Mucosal thickening	132(66)	61(30.5)	7(3.5)	0.000
Middle nasal concha pneumatization	81(61.4)	37(28.0)	14(10.6)	0.2
Sinus septa	61(47.3)	49(38.0)	19(14.7)	0.000

The mean IC-CR was 10.2, 10.7 and 11.4 mm in type 1, 2 and 3, respectively. Statistically significant difference was found between IC types and the IC-CR (*p* < 0.05). On the right side, the mean IC-CR was 10.6 mm in both males and females. On the left side, the mean IC-CR was 10.2 mm in females while it was 10.3 mm in males. There was no significant difference between the mean IC-CR and gender (*p* > 0.05) (Table 3).

**Table 3:** The relationship between IC-CR and localization and gender.

	IC-CR	
	Right	Left
	Mean (SD)	Mean (SD)
**Right protrusion**		
1	10.45 (2.4)	9.98 (2.3)
2	10.71 (2.4)	10.4 (2.1)
3	11.91 (3.1)	11.58 (2.9)
*p*-value	0.002	0.000
**Left protrusion**		
1	10.4 (2.4)	9.9 (2.2)
2	10.9 (2.6)	10.6 (2.4)
3	11.1 (2.4)	10.9 (2.8)
*p*-value	0.008	0.001
**Gender**		
Kadın	10.6 (2.4)	10.2 (2.2)
Erkek	10.6 (2.6)	10.3 (2.5)
*p*-value	0.7	0.5

The mean IC-MSW was 4.2 and 3.4 mm in females and males, respectively. The mean IOR-ICP was 10.5 and 11.2 mm in females and males, respectively. The mean IC-MSR were found to be 7.7 and 7.1 mm in females and males, respectively while the mean IC-MSF were found to be 28.3 and 27.0 mm in females and males, respectively. There was no significant difference between all measurements and gender (*p* > 0.05) (Table 4).

**Table 4:** The relationship between the measurements and gender.

	Gender			Z	*p*-value
		Number	Mean (SD)		
**IC-MSW**	Female	23	4.21 (2.9)	−1.5	0.1
	Male	33	3.4 (2.3)		
**IOR-ICP**	Female	23	10.5 (3.2)	−1.4	0.1
	Male	33	11.2 (2.5)		
**IC-MSR**	Female	23	7.7 (2.4)	−0.9	0.3
	Male	33	7.1 (2.1)		
**IC-MSF**	Female	23	28.3 (3.03)	T1 = .84	0.06
	Male	33	27.04 (3.3)		

## Discussion

This study reported a common degree of protrusion of type 1 IC. ICP is of clinical importance to be considered in terms of its localization, morphological variation and complications that may be caused in the relevant area.

Particularly in the fractures of zygomaticomaxillary complex, infraorbital nerve injury is commonly causes numbness of the midface and paresthesia. The rate of paresthesia and permanent sensory impairment in the innervation area of the infraorbital nerve in patients with maxillary fractures is 30%–80%.^16^, ^17^ In the cases of inferior orbital wall defects, there may be diplopia, paresthesia in the infraorbital nerve and ocular problems.^18^ The comprehensive knowledge of anatomy is essential for correct surgical operation to treat fracture cases. Other than the cause of fractures, hypoesthesia in the region that is innervated by the infraorbital nerve, has also been reported in neoplasia and orbital decompression surgery.^19^

During endoscopic sinus surgery, the length of the nerve opening to the maxillary sinus and the distance of the IC to the sinus roof should be considered. For example, during an extended endoscopic approach, if access to the infratemporal fossa is required, the posterior wall of the maxillary sinus must be removed and the infraorbital nerve will be at risk.^20^, ^21^ In this surgical operation, there is a narrowed field of view, which could otherwise lead to iatrogenic injuries and subsequent medico-legal problems.^22^

In individual cases where endonasal procedures are justified, complementary, localized transoral puncture of the maxillary sinus is recommended to remove hyperplastic mucosa in hidden anatomical regions.^23^, ^24^ In an unfavorable situation, a branch of the infraorbital nerve, particularly, the superior alveolar nerve is damaged.^25^ In 3/4 of all cases, complications such as swelling of the cheek, facial pain, numbness of the face or teeth and even paresthesia occur after surgery. In approximately 30% of patients, these complaints remain partially permanent, most likely as local dysesthesia.^24^

A relatively safe location for a complementary puncture is the intersection of two reference lines, i.e. a vertical line through the equilateral pupil and a horizontal line running exactly along the nasal floor.^26^ If these precautions are taken into account, the rate of temporary discomfort decreases to 45% and the rate of ongoing problems to 5%.^27^

There are different prevalence rates of ICP in the previous studies reported by Ference et al.^11^ (12.5%), by Kalabalık et al.^13^ (8.8%), by Lantos et al.^6^ (10.8%), Gautam et al.^28^ (11.4%), by Yenigun et al.^5^ (12.3%), and Haghnegahdar et al.^12^ (23.2%). Kalabalık et al.^13^ reported no significant difference in the prevalence of IC types either when comparing the right and left sides or males and females. In this study, although the prevalence of ICP (8%) was close to the study of Kalabalık et al.,^13^ it was generally found to be lower than other studies. Except type 1 and 3 of right side, no significant difference was found between the other types of right and left sides and gender.

Ference et al.,^11^ Kalabalık et al.^13^ and Fontolliet et al.^29^ reported that the most common type of IC as type 1 (60.5%, 55.2% and 68.5%, respectively). Açar et al.^2^ classified the ICP as 4 types, the first 3 types were the same as described in this study, the fourth type indicating the lateroantral course, and reported the most common type as type 1. In the study of Haghnegahdar et al.,^12^ the most common type was type 2 (50.3%). Li et al.^30^ also reported the most common IC type as type 2 (60%) in 10 adult cadaveric specimens. Gautam et al.^28^ and Lantos et al.^6^ classified ICP according to the length of the protruding component (Class 1 protrusion: 1‒3 mm, class 2: 4‒6 mm, class 3: 7‒11 mm^28^/≥7 mm^6^). Gautam et al.^28^ reported the percentage of class 1, 2 and 3 protrusion as 42%, 48% and 8.5% while Lantos et al.^6^ reported the corresponding values as 48%, 33% and 19%. In the present study, type 1 IC was found as the most common type.

In the study of Haghnegahdar et al.,^12^ in type 1, 2 and 3, the mean IC-CR was reported as 12.15, 12.35 and 12.92, respectively. In the study of Ference et al.,^11^ the infraorbital foramen was a mean distance of 11.99 mm lateral to the canine root. In this study, the mean IC-CR was 10.2, 10.7 and 11.4 mm in type 1, 2 and 3, respectively. Although the measurements in this study were found to be lower than the study of Haghnegahdar et al.,^12^ future studies will be guiding because, to the best of our knowledge, there is no another study related to this measurement in the literature.

For the measurements of type 3, Kalabalık et al.^13^ reported the mean IC-MSW, IOR-ICP, IC-MSR and IC- MSF as 3.75 mm, 9.51 mm, 6.76 mm and 25.44 mm, respectively. Ference et al.^11^ and Haghnegahdar et al.^12^ reported the IC-MSR as 8.58 mm and 11.61 mm, respectively. Gautam et al.^28^ and Lantos et al.^6^ reported that the median length of the protruding component along with the septum was 4.9 and 4 mm, respectively. IOR- ICP was found as 11 mm in the study of Lantos et al.^6^ Consistent with the literature, in this study, the mean IC-MSW, IOR-ICP, IC-MSR and IC-MSF was 3.8, 10.9, 7.4 and 27.7 mm, respectively. To the best of our knowledge, except for the study of Kalabalık et al.,^13^ another study measuring the ICP-MSF distance was not found in the literature.

To the best of our knowledge, only Ference et al.^11^ and Yenigun et al.^5^'s study has been found in the literature on the relationship of the IC types with variations in neighboring structures. In the study of Yenigun et al.,^5^ it was reported that concurrence of maxillary sinus septa and type 1 IC was found to be statistically significant on the right and left sides. There were no statistically significant correlations between any of the IC types and Haller cell and middle concha pneumatization on either side. Kalabalık et al.^13^ and Haghnegahdar et al.^12^ reported that the prevalence of Haller cells were found to be significantly higher in type 2 and 3 than in type 1. Ference et al.^11^ reported that ICP increased from 7.8% in cases without an ipsilateral Haller cell to 27.7% when a cell was present. In the study of Açar et al.,^2^ the correlation of Haller cell and maxillary sinus septa with IC types was not found statistically significant. In the present study, on the right and left side, statistically significant correlation was found between IC types and the presence of the Haller cell and sinus septa. But there was no significant correlation between IC types and middle concha pneumatization.

The different results in parameters, may be due to differences in IC types classification and in the method used (cadaver or radiological study) sample numbers, or may be due to racial or regional differences. Since there are few studies in the literature regarding the evaluation of some parameters in this study, it can be thought that this situation causes a limitation in the discussion part. However, we think that the new findings brought to the literature on this study subject will be a guide for future studies.

## Conclusion

Accurate diagnosis of ICP is very important in preventing infraorbital nerve damage in surgical procedures to be performed in the maxillary sinus region. In this study, it can be thought that the low percentage of cases where the IC completely protrudes into the maxillary sinus and the significant distance of the protruded IC to the floor of the maxillary sinus reduce the risk of nerve damage. However, in surgical procedures performed in locations close to the roof of the maxillary sinus, ignoring the ICP may cause undesirable results. Therefore, in all conditions, maximum attention should be paid to anatomical variations in surgical procedures.

## Ethical statements

The Ethics Committee of the Hatay Mustafa Kemal University approved this retrospective study.

## Funding

None.

## Conflicts of interest

The authors declare no conflicts of interest.
